# The effect of silencing 20E biosynthesis relative genes by feeding bacterially expressed dsRNA on the larval development of *Chilo suppressalis*

**DOI:** 10.1038/srep28697

**Published:** 2016-06-29

**Authors:** Jian Zhu, Yong-Cheng Dong, Ping Li, Chang-Ying Niu

**Affiliations:** 1 College of Plant Science & Technology, Huazhong Agricultural University, Wuhan 430070, China; 2 Pest Control Division, National Agricultural Technology Extension and Service Center, Ministry of Agricultural, Beijing 100125, China

## Abstract

RNA interference (RNAi) is a robust tool to study gene functions as well as potential for insect pest control. Finding suitable target genes is the key step in the development of an efficient RNAi-mediated pest control technique. Based on the transcriptome of *Chilo suppressalis*, 24 unigenes which putatively associated with insect hormone biosynthesis were identified. Amongst these, four genes involved in ecdysteroidogenesis *i.e., ptth*, *torso*, *spook* and *nm-g* were evaluated as candidate targets for function study. The partial cDNA of these four genes were cloned and their bacterially expressed dsRNA were fed to the insects. Results revealed a significant reduction in mRNA abundance of target genes after 3 days. Furthermore, knocked down of these four genes resulted in abnormal phenotypes and high larval mortality. After 15 days, the survival rates of insects in ds*spook,* ds*ptth*, ds*torso*, and ds*nm-g* groups were significantly reduced by 32%, 38%, 56%, and 67% respectively, compared with control. Moreover, about 80% of surviving larvae showed retarded development in dsRNA-treated groups. These results suggest that oral ingestion of bacterially expressed dsRNA in *C. suppressalis* could silence *ptth, torso, spook* and *nm-g*. Oral delivery of bacterially expressed dsRNA provides a simple and potential management scheme against *C. suppressalis.*

The rice stem borer, *Chilo suppressalis* Walker, is one of the most important rice pests in Asia, north Africa and southern Europe^1^. *C. suppressalis* larvae feed within plant stems, causing severe crop losses annually in rice-producing countries^2^. Currently, chemical control is being employed as the main strategy to manage this pest^3^. However, the excessive use of chemicals not only leads to insect resistance but also pollution^4^,^5^. Thus, there is an urgent need for alternative effective and environmental friendly strategy to be developed against this pest.

RNA interference (RNAi) is a technology that has become a potential robust tool for crop protection against insect pests^6^,^7^. It is a specific gene silencing mechanism mediated by double-stranded RNA (dsRNA), which has been harnessed as a useful reverse genetics tool in insects^8^. The selection of target genes is a key step to achieve pest control with RNAi^9^. Some genes associated with the signal pathways in insect development that could be used as potential targets for pest control using RNAi technology could be found from previous literature^10^,^11^,^12^,^13^.

Several key hormones and neuropeptides are involved in the control of insect developmental transitions, but the steroid hormone ecdysone (E) is the master regulator^14^. E is mainly synthesized in and released from the prothoracic glands (PGs) in larval stages^15^. Following secretion into the hemolymph, E is converted into an active form, 20-hydroxyecdysone (20E), which is the primary molting hormone^16^.

In the past decade, several genes crucial for the conversion of intermediates in ecdysteroid biosynthesis were successfully identified in other insects including *Drosophila melanogaster*^17^,^18^, *Bombyx mori*^19^ and *Manduca sexta*^20^ etc. The ecdysteroid biosynthesis in the prothoracic glands (PGs) begins with the conversion of cholesterol into 7-dehydrocholesterol (7dC), mediated by a Rieske oxygenase Neverland^21^. The next step between the 7dC and the first upstream compound exhibiting the highly characteristic ecdysteroid structure ketodiol, has not been fully understood and remains as the ‘Black Box’^22^,^23^. It is believed that the ‘Black Box’ contains the rate-limiting step in the production of ecdysone^24^. It is also supposed that the two genes involved with this step have been identified. A cytochrome P450 (CYP) enzyme, CYP307A1/A2 (Spook/Spookier)^25^,^26^ and a short-chain dehydrogenase/reductase, (Nm-g, in *Bombyx*/Sro, in *Drosophila*) have been reported to play a role in the ‘Black Box’^23^.

In addition, the ecdysteroid biosynthesis in PGs is mainly stimulated by a neuropeptide, known as the prothoracicotropic hormone (PTTH), which is produced by the brain neurosecretory cells^27^. PTTH activates ecdysteroidogenesis by binding to its receptor Torso, a receptor tyrosine kinase^28^. Previous studies showed that PTTH-dependent regulation might be expected to influence the ‘Black Box’ in 20E synthesis pathway^29^. It is likely that the PTTH signaling stimulates E production by targeting a component of the ‘Black Box’ and activating SPOOK^30^.

Therefore in this study, Illumina sequencing was used to sequence and analyze the transcriptome of *C. suppressalis* (whole body) aiming to identify the genes involved in regulating insect developmental transition, that is, three main hormones: 20E, PTTH and juvenile hormone (JH) biosynthesis related genes. Four of these genes including *ptth*, *torso*, *spook* and *nm-g* were selected for further studies using the RNAi technology. Bacterially expressed dsRNA of these 4 genes were fed to larval insects in an attempt to better understanding how these genes function in larval development and metamorphosis in *C. suppressalis*.

## Results

### Transcriptome overview

In order to obtain a comprehensive information about the genes of *C. suppressalis*, a library of the four developmental stages (eggs, larvae, pupae and adults) was constructed by Illumina sequencing platform. After filtering, approximately 100.6 million clean reads remained. A total of 46,603 unigenes were obtained with a mean size of 966 bp and N_50_ of 1,904 bp. About 24% of these unigenes were larger than 1,000 bp (Table 1).

**Figure 1:**
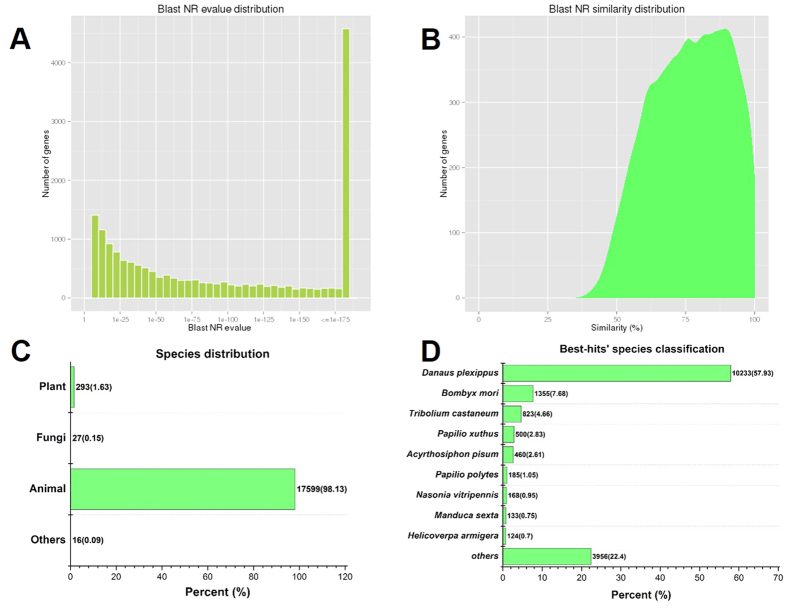
Characteristics of similarity search against Nr databases. (**A**) E-value distribution of BLAST hits for unigenes with a cutoff E-value of 1.0E^–5^. (**B**) Similarity distribution of the top BLAST hits for unigenes. (**C,D**) Organism species distribution of the top BLAST hits for unigenes in Nr database. The numbers of unigenes are indicated together with percentages placed in parentheses.

All unigene sequences were annotated by searching the Nr (NCBI non-redundant protein sequences) protein database using BLASTx with an E-value threshold of 10^−5^ (1.0E-5), a total number of 19,990 distinct sequences (43% of all unigenes) were matched with known genes that encoded for functional proteins. Based on E-value distribution of top hits, 60% of the mapped unigene sequences showed significant homology (less than 1.0E-50) with deposited genes in Nr database (Fig. 1A). By searching against the same database, 73% and 33% of the unigenes were found to possess sequence similarities greater than 50% and 75%, respectively (Fig. 1B). With respect to organism sources of homologs, almost 98% of unigenes with Nr annotations were matched to animal genes (Fig. 1C). While 58%, 8%, 5%, 3%, 3% of the mapped unigene sequences possess significant similarity, with orthologs *Danaus plexippus*, *Bombyx mori*, *Tribolium castaneum*, *Papilio xuthus* and *Acyrthosiphon pisum* respectively (Fig. 1D).

**Figure 2:**
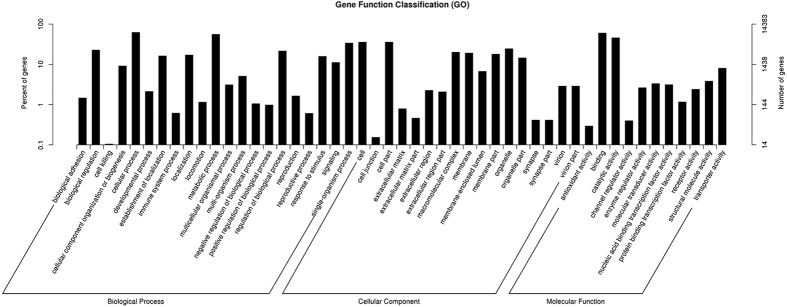
Gene ontology classifications of the *Chilo suppressalis* unigenes. The results were summarized in three main categories: biological process, cellular component, and molecular function. The right y-axis indicates the number of genes in the category. The left y-axis indicates the percentage of a specific category of genes in that main category.

For gene ontology (GO) analysis the 19,990 unigenes were divided into three ontologies, molecular functions, cellular components and biological processes. Amongst these, 14,383 unigenes (72%) were categorized into 49 functional groups. The largest group in each ontology was ‘cellular process’ (8,934, 62%), ‘cell’ (5,109, 36%) and ‘binding’ (8, 585, 60%) (Fig. 2).

### Genes involved in hormone synthesis and hormone receptors

Insect hormones (Juvenile hormone, 20-hydroxyecdysone and Prothoracictropic hormone) biosynthesis related transcripts were selected after gene annotation. By BLASTx analyses, a total of 24 putative genes were identified (Table 2), including 12 genes for JH biosynthesis and JH receptor (*acct*, *hmgs, hmgr, mevpk, mevk, mevppd, fpps, fpps-2, ippi, jhamt, met-1* and *met-2*), 10 genes for 20E biosynthesis and 20E receptor (*neverland, nm-g, spook, phm, dib, shade, shadow, ecr, ecr-A* and *usp)* and other two genes *ptth* and *torso* involved in PTTH signal pathway.

### Temporal and spatial transcript profiles

**Figure 3:**
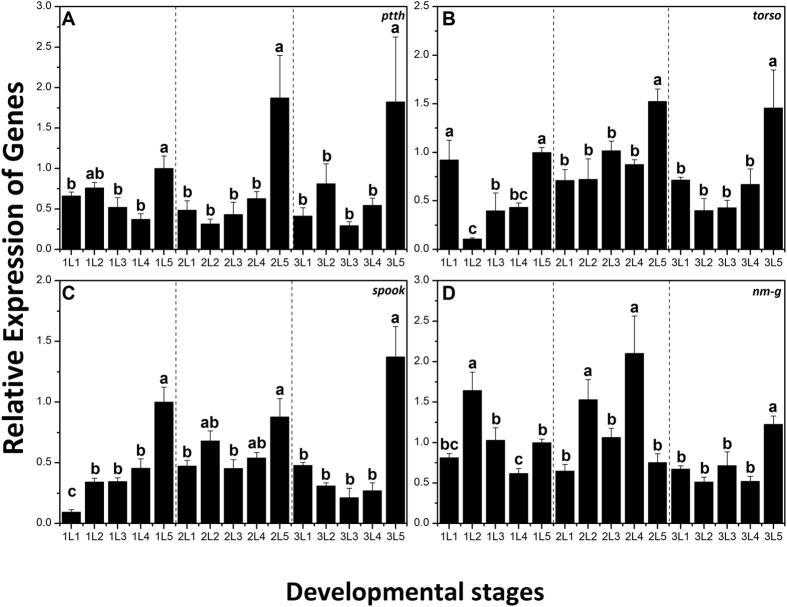
Relative expression levels of *ptth* (A), *torso* (B), *spook* (C), *nm-g* (D) for different differential stages of *Chilo suppressalis*. Expression levels at 15 different time points in first-instar and second-instar larvae were detected by q-PCR. Different letter shows significant difference (P < 0.05, ANOVA).

Based on analyzing and preliminary screening of the aforementioned genes, four genes (*ptth*, *torso*, *spook* and *nm-g*) were selected for the characterization of their expression patterns during different developmental stages (early and late 1^st^ to 3^rd^ instar larvae) by q-PCR. In general, all of the four genes displayed similar developmental expression patterns, with the highest expression level at the last day of each instar and kept a stable expression level in other days except *nm-g* in the 1^st^ and 2^nd^ instar larvae (Fig. 3).

**Figure 4:**
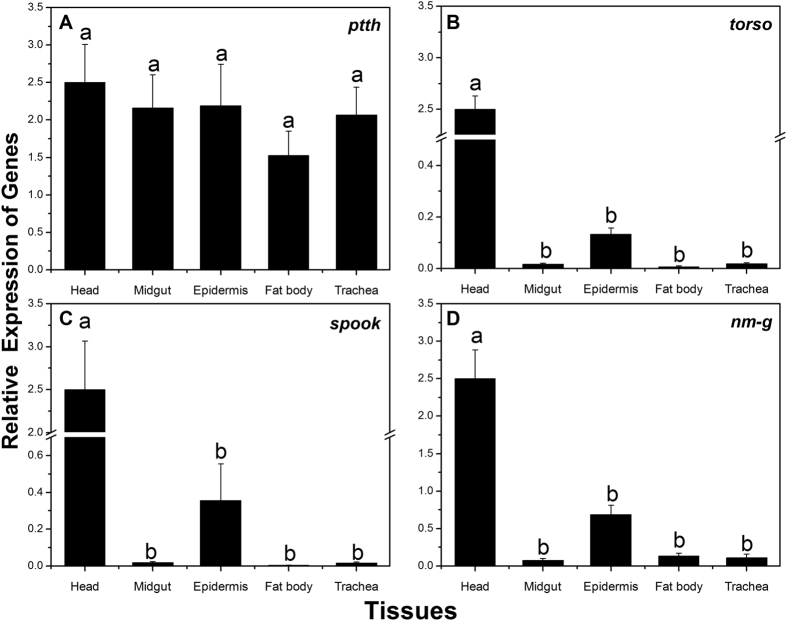
Relative expression levels of *ptth* (A), *torso* (B), *spook* (C), *nm-g* (D) in different tissues of the 3^rd^ instar larvae of *Chilo suppressalis*. Expression levels in the head, midgut, cuticula, fat body, and trachea were detected by q-PCR. Different letter shows significant difference (P < 0.05, ANOVA).

The spatial distribution of these four genes in different tissues (head, midgut, cuticula, fat body and trachea) of 5 days old 3^rd^ instar (3L5) larvae were also observed. The results showed that the four target genes were expressed in all five tissue types, however, gene expression patterns differed within tissue types. All of the four genes were highly expressed in the head, and there was a significant difference between head and other tissue types excluding *ptth,* which has abundant expression in all of the five tissues (P < 0.05) (Fig. 4).

### The relative expression of *ptth, torso, spook, nm-g* after RNAi-treatment

**Figure 5:**
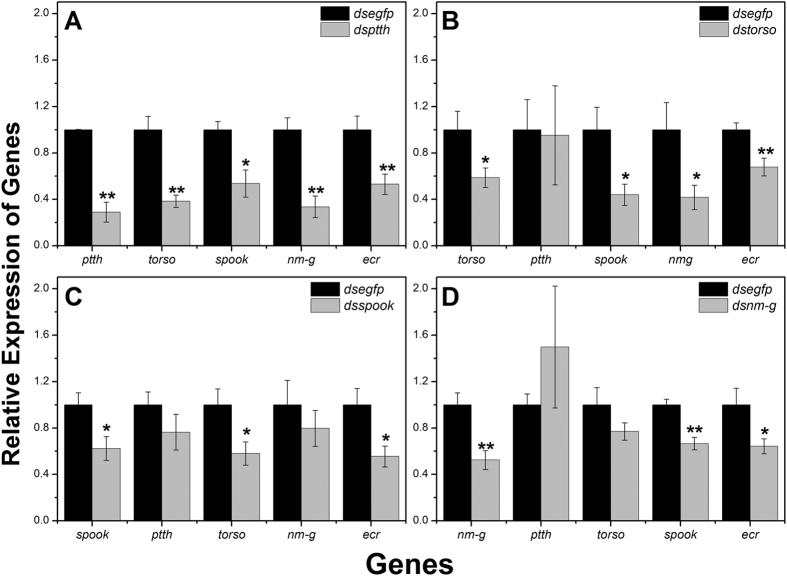
Relative mRNA abundance of the five 20E synthesis genes after RNAi-treated with ds*ptth* (A), ds*torso* (B), ds*spook* (C) and ds*nm-g* (D). The mRNA levels of genes were monitored by q-PCR at 72 h after feeding. Larvae fed with ds*egfp* were used as negative control. Asterisk shows significant difference (*p < 0.05 and **p < 0.01, Student’s *t*-test).

After feeding insects with diets containing dsRNA for 3 days, larvae were randomly sampled to examine the mRNA abundance of *ptth*, *torso*, *spook* and *nm-g* by q-PCR. The mRNA levels of these genes in treated larvae were significantly reduced by 71%, 46%, 40%, 35%, respectively, compared with the control, ds*egfp* (enhanced green fluorescent protein) treatments (P < 0.05) (Fig. 5).

The knockdown of *ptth* significantly blocked the transcript accumulation of other three genes (P < 0.05) (Fig. 5A). Knockdown of *torso* significantly suppressed the expression of *spook* and *nm-g* but did not suppress mRNA level of *ptth* (P < 0.05) (Fig. 5B). The knockdown of *spook* effectively suppressed the relative expression of *torso*. Similarly, feeding on ds*nm-g* only blocked the transcript level of *spook* (P < 0.05) (Fig. 5C,D).

In addition, we also examined the transcript level of *ecr,* which was one of 20E heterodimeric nuclear receptors and was regulated by 20E through a positive feedback loop in *D. melanogaster.* As expected, *ecr* was significantly reduced in all four treatment groups.

### Effect of dsRNA on larval survival

**Figure 6:**
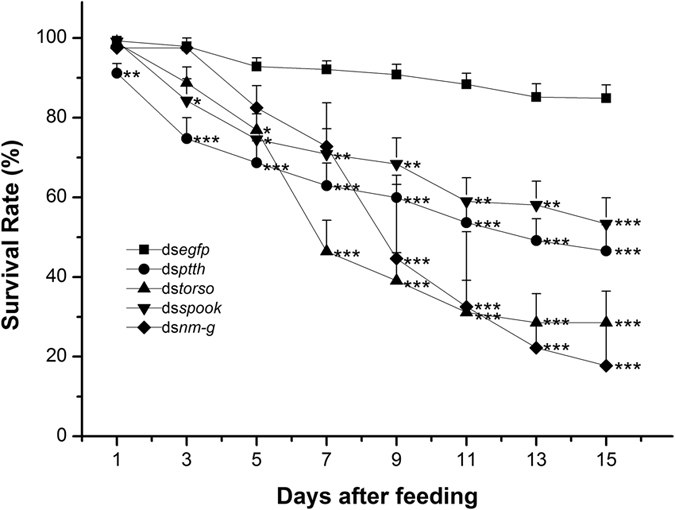
The change of larval survival of *Chilo suppressalis* after dietary ingestion ds*egfp*, ds*ptth*, ds*torso*, ds*spook*, ds*nm-g*. The insects were continuously exposed to dsRNA through 3-day-old larvae. Larvae fed with d*segfp* were used as negative control. Asterisk shows significant difference (*p < 0.05, **p < 0.01 and ***p < 0.001, Student’s *t*-test).

Survival rates in RNAi-treated groups were calculated for each gene 1 to 15 days after treatment. Individual insects that did not move after touching with a camel’s hair brush were considered dead. In all treatments, the survival rate of RNAi-treated groups reduced over time. On the 9^th^ day, a significantly lower survival rate was observed in the target dsRNA treated groups (P < 0.05). After 15 days, the survival rates of these treatments reduced to 38%, 56%, 32% and 67% in ds*ptth*, ds*torso*, ds*spook* and ds*nm-g* groups respectively when compared with the control group (ds*egfp*-ingested larvae) (Fig. 6).

### Retarded development and abnormal phenotypes

**Figure 7:**
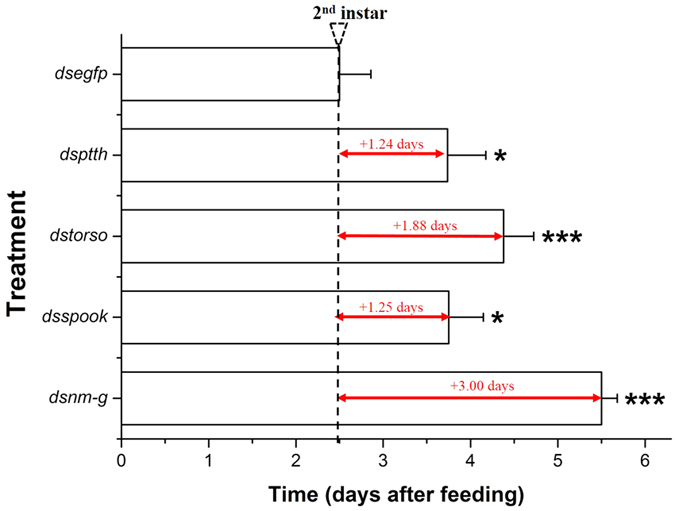
The different developmental period among RNAi-treated groups. Larvae fed with ds*egfp* were used as negative control. Asterisk shows significant difference (*p < 0.05, **p < 0.01 and ***p < 0.001, Student’s *t*-test).

**Figure 8:**
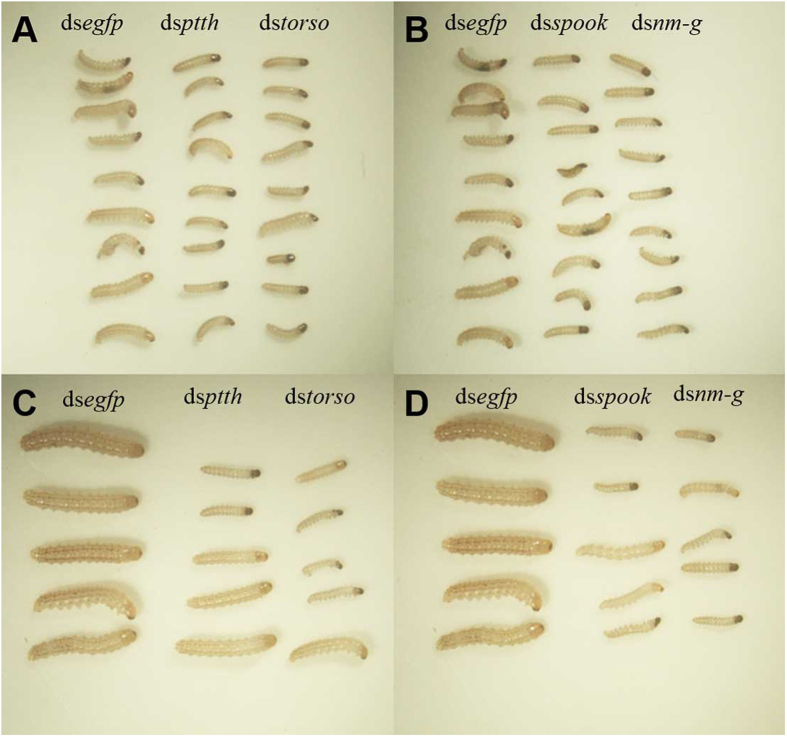
Retarded development in RNAi-treated groups. The surviving larvae in each RNAi-treated group showed retarded development. The group fed with ds*egfp* was used as the negative control. (**A,B**) were taken 2 days after feeding, (**C,D**) were taken after 7 days after feeding.

**Figure 9:**
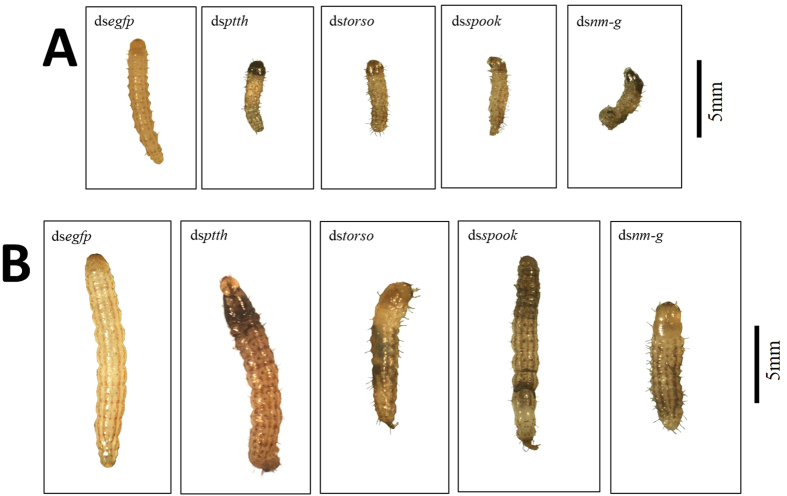
Developmental defects in RNAi-treated individuals. The dead larvae in each RNAi-treated group showing abnormal phenotypes during metamorphosis. The group fed with ds*egfp* was used as the negative control. (**A**) represents metamorphosis from 1^st^ to 2^nd^ instar larvae. (**B**) represents metamorphosis from 2^nd^ to 3^rd^ instar larvae.

Our results revealed that the larval developmental time was significantly extended in the target dsRNA-treated groups (P < 0.05) (Fig. 7). In addition, about 80% of surviving larvae in the RNAi-target treated groups showed retarded development (Fig. 8). Almost all dead larvae had abnormal phenotypes, such as “half-ecdysis” during the molting process, ‘melanism’ in head or thorax. (Fig. 9).

## Discussion

In this study, *C. suppressalis* whole body transcriptome analysis was performed with the objective of identifying target genes which could be exploited for future pest management programs in addition to providing a valuable genetic resource. In the past, transcriptome analysis of *C. suppressalis* were carried out using specific tissues like the midgut^31^, antennal^32^ and ovipositor-pheromone gland^33^. However, comparative of these transcriptomic data showed that present study have longer unigenes (mean length = 966 bp) and deeper coverage of transcripts (about 10 GB).

Four selected genes (*ptth*, *torso*, *spook* and *nm-g*) involved with ecdysteroidogenesis were cloned and characterized. Observation of their spatial and temporal transcript profiles revealed that *torso*, *spook* and *nm-g* clearly had a high transcript level in the head where PGs are located. Previous studies on tissues specificity expression of *spook* and *nm-g* in *Drosophila melanogaster* (*spook* and *nm-g*)*, Bombyx mori* (*spook* and *nm-g*) *Schistocerca gregaria* (*spook*) and *Spodoptera littoralis* (*spook*) showed mostly expression in the ring gland or in the PG during larval stage^23^,^25^,^34^,^35^. On the other hand, *ptth* was expressed in all the five tissues with no significant difference in its expression between the tissues (Fig. 4). Similar expression of the *ptth* in different larval tissues has been reported in other lepidopteron insects. For example in *Helicoverpa armigera*, the brain is the major site for *ptth* expression and low levels of expression were also reported in the midgut and fat body^36^. *ptth* has also been detected in different tissues of *Sesamia nonagrioides*, including brain, ganglion, gut and fat body^37^, however, the function of PTTH produced in other organs still remains unclear. Furthermore, we found that the four genes showed three expression peaks in late 1^st^, 2^nd^ and 3^rd^ instar stages (Fig. 3). These peaks were clearly reduced in the newly-molted 2^nd^ and 3^rd^ instar larvae. This expression pattern is similar to those reported in other insects^23^,^34^,^38^,^39^,^40^. In addition, it has been reported that the expression pattern of a gene which has an inerratic change around ecdysis, is almost synchronized with that ecdysone titer in insects^41^. Thus, the spatial and temporal expression patterns suggest that these four genes might be directly involved in the ecdysteroidogenesis in *C. suppressalis.*

This study reveals that RNAi-mediated depletion of *ptth, torso, spook* and *nm-g* in *C. suppressalis* could suppress their transcriptional levels, resulting in significant growth retardation (Figs 7 and 8) and lethal phenotypes (Fig. 9). Moreover, the phenotypic defects are similar to insects whose ecdysteriod synthesis had been disturbed^10^,^39^,^42^ or whose ecdysteriod-mediated signaling had been inhibited^43^,^44^. Retarded growth was also reported in the larvae of *Drosophila* with ablated PG neurons^45^ and *nm-g* RNAi mediated gene silencing^23^. In addition, RNAi-mediated silencing of the four target genes reduced the ecdysone receptor gene *ecr* expression at mRNA levels. Similarly, it has also been reported in other insects that gene mutations and/or RNAi mediated gene silencing of Halloween enzymes caused a decrease in ecdysteroid titers^34^,^46^. In addition, the expression of *ecr* is regulated by ecdysteroids through a positive feedback loop in *D. melanogaster*^47^. Accordingly, our results raised the possibility that dietary introduction of ds*ptth*, ds*torso,* ds*spook* and ds*nm-g* inhibit ecdysteroid signaling pathway.

It is very important to understand the cross-talk between the PTTH signaling pathway and 20E synthesis pathway. We assumed that when PTTH bind to *torso* receptor, it triggers E and 20E biosynthesis in PG. This PTTH-dependent regulation might be expected to influence the ‘Black Box’. In this work, after the knockdown of one target gene, the transcription levels of other three genes were examined. It was observed in both ds*ptth* and ds*torso* groups, the genes *nm-g* and *spook* were down-regulated substantially in response to RNA-mediated silencing of *ptth* and *torso*. However, there was no significant change of *ptth* and *torso* in both ds*spook* and ds*nm-g* groups except *torso* in ds*spook* group. This indicates that the knockdown of *spook* was partially suppressed by the induction of *torso*. Recently, it has been found that PTTH has a positive effect on the transcription of genes directly involved in the E biosynthetic pathway i.e. *spook, phantom, disembodied* and *shadow* in both *Bombyx* and *Drosophila*^48^,^49^. In *Drosophila*, *sro* expression was significantly reduced in the third instar larvae in which the *ptth* gene-expressing neurons were ablated^23^. A similar finding was observed in this study which suggests ‘Black Box’ is under the regulation of PTTH. It also indirectly proves that SPOOK is the target of PTTH signaling^30^. In addition, the knockdown of *ptth* significantly suppressed *torso*, but there was no change of *ptth* by the knockdown of *torso*. This could be because *torso* is specifically expressed in head whereas *ptth* is not only expressed in head but also in other tissues. The same pattern of expression is shown by *nm-g* and *spook* as previously explained for *ptth* and *torso*. This result suggests that the *spook* is the downstream gene of *nm-g*^23^.

In the past, microinjection was used to administer dsRNA into insects in an attempt to study their gene function. Although this technique was successful, however, it had a lot of drawback including high cost and short duration of synthetic dsRNA *in vivo*^50^. Therefore the oral administration of bacterially expressed dsRNA presents a more convenient and applicable strategy against pests. In recent years this method has successfully been used to study the gene function in variety of insects^51^,^52^,^53^. This study presents for the very first time the use of bacterially expressed dsRNA fed to insects in an attempt to explore the functional genes of an important agricultural pest, *C. suppressalis*. Therefore, the screening of these four key genes will provide a base for future silencing/knock-out technique applications such as plant-mediated RNAi^54^ and CRISPR/Cas9^55^, which leads to sustainable management of insect pests in the long run.

## Conclusion

The transcriptome of *C. suppressalis* was sequenced with Illumina that yielded 46,603 unigenes. Of these, 24 putative genes involved in hormone synthesis and hormone receptors were identified. This data adds a substantial contribution to the existing sequence resources for this important rice pest and provides a comprehensive knowledge for potential molecular targets in the control of *C. suppressalis*. In addition, *ptth*, *torso*, *spook* and *nm-g* play an essential role in regulating ecdysteroidogenesis during the development and metamorphosis in *C. suppressalis*. These four genes could be potential targets for an effective management of this pest. The present study suggests that oral ingestion of dsRNA which target key specific genes may develop a novel control strategy for *C. suppressalis*. Our future work will focus on functional studies of key genes related to JH biosynthesis and the cross-talk among 20E, JH and PTTH in *C. suppressalis*.

## Materials and Methods

### Insects sample

Insects used in this study were maintained in the laboratory on artificial rice shoots, sugar, yeast and soy bean at 28 °C ± 2 under a long photoperiod (16L-8D) and >80% relative humidity. RNA was extracted from eggs, larvae (different ages), pupae (early and late) and adults. After extraction, RNA samples were immediately frozen in liquid nitrogen and maintained at −80 °C until further use.

### RNA sequencing

Prior to sequencing, a 1% agarose gel was used to check for degradation and contamination of RNA samples, in addition, the purity and quality of the RNA samples were checked using Nanodrop Spectrophotometer (IMPLEN, CA, USA) and RNA Nano 6000 Assay Kit of the Agilent Bio-analyzer 2100 system (Agilent Technologies, CA, USA) respectively. A total of 3 μg from each sample was used as a template for RNA preparation summarized as follows: Poly-T oligo attached magnetic beads were used for mRNA purification followed by fragmentation and synthesis of the first and second strands of cDNA. The preferential 150–200 bp cDNA fragment was selected and purified with AMPure XP system (Beckman Coulter, Beverly, USA). A size selected adaptor was ligated to cDNA followed by PCR. Finally PCR products were purified and library quality was determined on the Agilent Bioanalyzer 2100 system. Illumina Hiseq 2000 platform sequencing was carried out after cluster generation and library preparations. About 100 bp Paired-end reads were generated.

### Analysis of transcriptome data

First Illumina data was processed using in-house pearl script and cleaned reads were obtained after low quality reads, poly-N reads and adapter were removed from raw data. Simultaneously, the clean data was used to estimate Q20, Q30, GC-content and the sequencing duplication level. From this point clean reads with high quality were used for subsequent data analysis. The left (read1 files) and right (read2 files) files from all libraries/samples were pooled into two different big left.fq file and right.fq file respectively. These big files were used to accomplish transcriptome assembly using Trinity^56^,^57^ with all parameters set to default and min_kmer_cov set to 2 by default. Then, unigenes were blasted against NCBI nucleotide collection (nr) database with an E value cutoff of 10^−5^ (E-value < 10^−5^). In an attempt to obtain proteins with highest sequence similarity to the given unigenes and their putative functional annotation, the unigene sequences were also aligned by BLASTx to protein databases such as Swiss-Prot, KEGG, and COG. Finally gene function was annotated based on the results obtained from the different databases including Pfam, KO, GO, Nr, Nt, KOG/COG and Swiss-Prot.

### RNA isolation and cDNA synthesis

Insects were frozen in nitrogen and homogenized with a tissue grinder. Total RNA was extracted from these insect samples using Trizol (Invitrogen, Carlsbad, CA) reagent according to manufacturer’s instructions. Agarose gel electrophoresis and spectrophotometer electrophoresis were used to check the quality and concentration of the RNA samples followed by digestion with DNase 1 to eliminated genomic DNA contaminations. cDNA synthesis was carried out in triplicates by reversed transcription in 20 μl reactions containing 50 mM of oligo (dT) primer, ~1 μg of total RNA.

### Vector construction and dsRNA preparation of *ptth, torso, spook* and *nm-g* genes

In order to construct the plasmid that expresses the dsRNA of *ptth, torso, spook* and *nm-g*, fragments of each of these 4 genes were amplified using their specific primers (Table 3). And *egfp* (enhanced green fluorescent protein) fragment which was used as control was amplified from Pub·nls·EGFP. PCR were carried out under the following conditions: 95 °C for 30 s, 56 °C for 30 s, and 72 °C for 60 s for 30 cycles and a final extension step of 72 °C for 10 min. PCR products were confirmed by electrophoresis on 1% agarose gel and purified with DNA gel extraction kit (Axygene, USA). PCR products were cloned into pMD18T vector with Kpn I and Hind III sites and later transformed into DH5α competent cells. Positive clones containing the 5 different plasmids designated as pMD-18T-*ptth*, pMD-18T-*torso*, pMD-18T-*spook*, pMD-18T-*nm-g* and pMD-18T-*egfp* were sent to Invitrogen-China for sequencing to ensure insertion. After sequencing the 5 different plasmids were digested from pMD-18-T vector and cloned to L4440 vector then the recombinant vectors L4440-*ptth*, L4440-*torso*, L4440-*spook*, L4440-*nm-g* and L4440-*egfp* were transformed into the RNaseIII deficient *E. coli* strain HT115 (DE3), which is unable to degrade dsRNA. dsRNA single colonies of HT115 (DE3) bacteria containing 4 different plasmids were produced by growing these bacteria in LB for 14 h at 37 °C with shaking under antibiotics selection (100 mg/ml ampicillin). The culture was diluted 100-fold in 2 × YT medium and allowed to grow to OD_595_ ≈ 0.5. Synthesis of T7 polymerase was induced by adding 0.5 mM of isopropyl β-D thiogalactopyranoside (IPTG) to bacteria and incubating again for 4 h at 37 °C. The dsRNA of the 5 different plasmids was extracted as described above and their length was confirmed by electrophoresis on a 1% agarose gel. To prepare bacterial cells that express dsRNA, bacterial cells were collected from 1,000 ml IPTG-induced culture by centrifuging at 4,000 rpm for 5 mins and then re-suspended in 4 ml sterile water for *C. suppressalis* feeding bioassays.

### dsRNA feeding bioassays

Prior to feeding, artificial diet was cut into rectangular pellets (5 mm × 5 mm × 3 mm) weighing about 0.2 g. Each pellet was coated with a solution containing re-suspended bacteria expressing dsRNA and fed to 3-days-old *C. suppressalis* larvae. All diets were replaced daily. Insect molting, survival, and abnormal phenotypes were observed and recorded daily. Data on survival rate and developmental duration were analyzed by Student’s *t*-test. All samples were photographed using a Nikon DS-Fi1 digital camera. In addition, 3 days after feeding some larvae were collected from each treatment, frozen in liquid nitrogen and kept at −80 °C. These samples were used for RNA extraction. Extracted RNA was later used for q-PCR to test for the expression of target genes.

### Quantitative Real Time-PCR Validation

Total RNA samples were collected from the head, midgut, cuticula, fat body and trachea in 3L5 larvae for spatial transcript profile; every day from 1L1 larvae to 3L5 larvae for temporal transcript profile and from larvae subjected to 3-day’s bioassays for testing the relative expression of genes after RNAi-treatment. All samples were extracted by using Trizol reagent (Invitrogen) according to the manufacturer’s instructions. Each sample contained 5 larvae and replicated thrice. The house-keeping gene *EF-1* was used as internal control^58^. The primers of target genes were designed with Primer premier 5 (Table 3). The expression profiles of the four genes were determined using q-PCR with ABI 7300 Real-Time PCR detection system (Applied Biosystems Inc, Foster City, CA, USA). The PCR program included 95 °C for 30 s, followed by 40 cycles of 95 °C for 5 s, 55 °C for 30 s and 72 °C for 31 s. The total reaction volume was 20 μl. All reactions were performed in triplicates.

### Statistical analysis

Data analysis was carried out using SPSS 16.0 (SPSS Inc., Chicago, Illinois, USA) and Origin 9.0 (Electronic Arts Inc., Redwood, California, USA) software. Temporal and spatial transcript profiles were analyzed with one-way ANOVA followed by a Tukey HSD multiple range test. Student’s *t*-test was used to compare the relative expression of genes and the survival rate between the RNAi-treatments after dsRNA feeding. The q-PCR data were analyzed using the delta-delta Ct method.

## Additional Information

**How to cite this article**: Zhu, J. *et al*. The effect of silencing 20E biosynthesis relative genes by feeding bacterially expressed dsRNA on the larval development of *Chilo suppressalis*. *Sci. Rep.* **6**, 28697; doi: 10.1038/srep28697 (2016).

## Figures and Tables

**Table 1:** Summary statistics of transcriptome of *Chilo suppressalis*.

**Summary statistics**	
Raw Reads	104,288,138
Clean Reads	100,748,860
Number of unigenes	46,603
Mean length of unigenes (bp)	966
N_50_ of unigenes (bp)	1,904
Length of unigenes >1,000 bp	12,777
Sequences with E-value < 10^–5^	19,990

**Table 2:** Putative genes involved in insect hormone synthesis and hormone receptors in *Chilo suppressalis.*

**Name**	**Gene ID**	**Length(bp)**	**Name**	**Acc.number**	**Species**	**E value**	**Identity (%)**
Juvenile hormone synthase relative genes and its receptor							
aact	comp45531_c0	1766	acetoacetyl-CoA thiolase	AB274988.1	[*Bombyx mori*]	0.0E + 00	84
hmgs	comp36923_c0	2565	3-hydroxy-3-methylglutaryl-CoA synthase	AB274989.1	[*Bombyx mori]*	0.0E + 00	87
hmgr	comp40204_c0	4386	3-hydroxy-3-methylgluraryl coenzyme A reductase	AJ009675.1	[*Agrotis ipsilon*]	0.0E + 00	88
mevpk	comp43114_c0	1346	phosphomevalonate kinase	AB274992.1	[*Bombyx mori]*	3.2E − 76	65
mevk	comp43915_c0	2916	mevalonate kinase	AB274991.1	[*Bombyx mori*]	0.0E + 00	76
mevppd	comp38764_c0	1654	diphosphomevalonate decarboxylase	AB274993.1	[*Bombyx mori*]	0.0E + 00	71
fpps-2	comp6260_c0	509	farnesyl diphosphate synthase 2	AB274996.1	[*Bombyx mori*]	2.3E − 30	47
fpps	comp37620_c0	1951	farnesyl diphosphate synthase	AY954921.1	[*Mythimna unipuncta*]	0.0E + 00	81
ippi	comp35679_c1	1745	isopentenyl-diphosphate delta isomerase	AK401537.1	[*Papilio xuthus*]	5.6E − 153	84
jhamt	comp22657_c2	919	juvenile hormone acid methyltransferase	DQ465408.1	[*Samia ricini*]	1.9E − 41	55
met-1	comp17057_c0	1584	methoprene-tolerant homolog-1	AB359911.1	[*Bombyx mori*]	0.0E + 00	75
met-2	comp131119_c0	2385	methoprene-tolerant homolog-2	AB359912.1	[*Bombyx mori*]	0.0E + 00	49
20-hydroxyecdysone synthase relative genes and its receptor							
neverland	comp3754_c0	685	Rieske-domain protein neverland	GU391576.1	[*Spodoptera littoralis*]	1.8E − 106	73
spook	comp40205_c0	2278	cytochrome P450 CYP307A1	FJ981602.1	[*Spodoptera littoralis*]	0.0E + 00	79
nm-g	comp24683_c0	856	short-chain dehydrogenase	AK402634.1	[*Papilio polytes*]	8.9E − 144	63
pha	comp39538_c0	1738	cytochrome P450 CYP306A1	FJ010194.1	[*Spodoptera littoralis*]	0.0E + 00	80
dib	comp12865_c0	1667	cytochrome P450 CYP302A1	DQ357067.1	[*Manduca sexta*]	0.0E + 00	66
shade	comp39121_c1	1857	cytochrome P450 CYP314A1	DQ372988.1	[*Manduca sexta*]	0.0E + 00	80
shadow	comp36484_c0	1630	cytochrome oxidase	AK401875.1	[*Papilio xuthus]*	2.8E − 127	74
ecr	comp44265_c0	4146	ecdysone receptor	XM_002427373.1	[Pediculus humanus corporis]	6.7E − 171	60
ecrA	comp42790_c0	4045	ecdysone receptor A isoform	AB067811.1	[*Chilo suppressalis*]	0.0E + 00	98
usp	comp43786_c1	4118	Ultraspiracle	AB081840.1	[*Chilo suppressalis*]	0.0E + 00	100
Prothoracicotropic hormone synthase relative genes and its receptor							
ptth	comp18067_c0	413	prothoracicotropic hormone PTTH	AY172671.1	[*Heliothis virescen*s]	2.9E − 38	52
torso1	comp265585_c0	424	tyrosine-protein kinase receptor torso	NM_001170578.1	[*Bombyx mori*]	1.2E − 62	69

**Table 3:** Primers used in the experiments.

**Primer**	**Sequence(5′-3′)**
Primer used in q- PCR	
q-ptth(F)	GCGATGGCTTTGAAGAA
q-ptth(R)	TCCGCTGCCTTGATTAC
q-torso(F)	CAGAAAGGCAATGGCAAGA
q-torso(R)	TCACCACGCCGAACGAC
q-spook(F)	GAACTCCCATTGCGACTC
q-spook(R)	CTCTTGCCGATGCTGAA
q-nm-g(F)	GGCAGGCTTACTGGGTTGG
q-nm-g(R)	TGGGTGGTTTGGTTCGACATA
q-ecr(F)	CGGCCAGGACTGGAACA
q-ecr(R)	AAGGAACGGCGGCAACT
q-EF-1(F)	TGAACCCCCATACAGCGAATCC
q-EF-1(R)	TCTCCGTGCCAACCAGAAATAGG
Synthesizing the dsRNA	
ptth(F)	CTTCCCACCAAGGTCACCTTGG
ptth(R)	GGTGTATCCGGTATGGCTTG
torso(F)	GCGACCTGCTCACATACC
torso(R)	TCTTGCCATTGCCTTTCT
spook(F)	AGGACATCCGCACCTTCAT
spook(R)	TCTCGGGTTCCCTCCAGTA
nm-g(F)	GTTTGGTCACCGTTGCTG
nm-g(R)	TCGCCACATTTATTACTCG
egfp(F)	CCCTGAAGTTCATCTGCACC
egfp(R)	GTGCTCAGGTAGTGGTTGTC
